# Contribution of rare variant associations to neurodegenerative disease presentation

**DOI:** 10.1038/s41525-021-00243-3

**Published:** 2021-09-28

**Authors:** Allison A. Dilliott, Abdalla Abdelhady, Kelly M. Sunderland, Sali M. K. Farhan, Agessandro Abrahao, Malcolm A. Binns, Sandra E. Black, Michael Borrie, Leanne K. Casaubon, Dar Dowlatshahi, Elizabeth Finger, Corinne E. Fischer, Andrew Frank, Morris Freedman, David Grimes, Ayman Hassan, Mandar Jog, Sanjeev Kumar, Donna Kwan, Anthony E. Lang, Jennifer Mandzia, Mario Masellis, Adam D. McIntyre, Stephen H. Pasternak, Bruce G. Pollock, Tarek K. Rajji, Ekaterina Rogaeva, Demetrios J. Sahlas, Gustavo Saposnik, Christine Sato, Dallas Seitz, Christen Shoesmith, Thomas D. L. Steeves, Richard H. Swartz, Brian Tan, David F. Tang-Wai, Maria C. Tartaglia, John Turnbull, Lorne Zinman, ONDRI Investigators, Robert A. Hegele

**Affiliations:** 1 Robarts Research Institute, Schulich School of Medicine and Dentistry, Western University, London, ON Canada; 2 Department of Biochemistry, Schulich School of Medicine and Dentistry, Western University, London, ON Canada; 3 Department of Biology, Schulich School of Medicine and Dentistry, Western University, London, ON Canada; 4 Rotman Research Institute, Baycrest Health Sciences, Toronto, ON Canada; 5 Departments of Neurology and Neurosurgery, and Human Genetics, Montreal Neurological Institute, McGill University, Montreal, QC Canada; 6 Division of Neurology, Department of Medicine, Sunnybrook Health Sciences Centre and University of Toronto, Toronto, ON Canada; 7 Dalla Lana School of Public Health, University of Toronto, Toronto, ON Canada; 8 LCCampbell Cognitive Neurology Research Unit, Hurvitz Brain Sciences Research Program Sunnybrook Health Sciences Research Program, Sunnybrook Health Sciences Centre, Toronto, ON Canada; 9 St. Joseph’s Health Care Centre, London, ON Canada; 10 Schulich School of Medicine and Dentistry, Western University, London, ON Canada; 11 Department of Medicine, Division of Neurology, University of Toronto, Toronto, ON Canada; 12 University Health Network Stroke Program, Toronto Western Hospital, Toronto, ON Canada; 13 Department of Medicine, University of Ottawa, Ottawa, ON Canada; 14 Ottawa Hospital Research Institute, Ottawa, ON Canada; 15 Department of Clinical Neurological Sciences, Schulich School of Medicine and Dentistry, Western University, London, ON Canada; 16 Lawson Health Research Institute, London, ON Canada; 17 Keenan Research Centre for Biomedical Research, Li Ka Shing Knowledge Institute, St. Michael’s Hospital, Toronto, ON Canada; 18 Bruyère Research Institute, Ottawa, ON Canada; 19 Division of Neurology, Department of Medicine, Baycrest Health Sciences, Mt. Sinai Hospital and University of Toronto, Toronto, ON Canada; 20 Thunder Bay Regional Research Institute and Northern Ontario School of Medicine, Thunder Bay, ON Canada; 21 London Health Sciences Centre, London, ON Canada; 22 Campbell Family Mental Health Research Institute, Centre for Addiction and Mental Health, Toronto, ON Canada; 23 Department of Psychiatry, Faculty of Medicine, University of Toronto, Toronto, ON Canada; 24 Centre for Neuroscience Studies, Queen’s University, Kingston, ON Canada; 25 Edmond J. Safra Program in Parkinson’s Disease and the Morton and Gloria Shulman Movement Disorders Clinic, Toronto Western Hospital, Toronto, ON Canada; 26 Cognitive & Movement Disorders Clinic and L.C. Campbell Cognitive Neurology Research Unit, Hurvitz Brain Science Program, Sunnybrook Health Sciences Centre, Toronto, ON Canada; 27 Cognitive Neurology and Alzheimer’s Disease Research Centre, Parkwood Institute, St. Joseph’s Health Care, London, ON Canada; 28 Department of Psychiatry and Toronto Dementia Research Alliance, University of Toronto, Toronto, ON Canada; 29 Tanz Centre for Research in Neurodegenerative Diseases, University of Toronto, Toronto, ON Canada; 30 Department of Medicine, McMaster University, Hamilton, ON Canada; 31 Li Ka Shing Knowledge Institute, St. Michael’s Hospital, Toronto, ON Canada; 32 Clinical Outcomes and Decision Neuroscience Unit, St. Michael’s Hospital, University of Toronto, Toronto, ON Canada; 33 Cumming School of Medicine, University of Calgary, Calgary, AB Canada; 34 Division of Neurology, St. Michael’s Hospital, Toronto, ON Canada; 35 Krembil Research Institute, Toronto Western Hospital, Toronto, ON Canada; 36 University Health Network Memory Clinic, Toronto Western Hospital, Toronto, ON Canada

**Keywords:** Genetic association study, Neurodegenerative diseases, Targeted resequencing

## Abstract

Genetic factors contribute to neurodegenerative diseases, with high heritability estimates across diagnoses; however, a large portion of the genetic influence remains poorly understood. Many previous studies have attempted to fill the gaps by performing linkage analyses and association studies in individual disease cohorts, but have failed to consider the clinical and pathological overlap observed across neurodegenerative diseases and the potential for genetic overlap between the phenotypes. Here, we leveraged rare variant association analyses (RVAAs) to elucidate the genetic overlap among multiple neurodegenerative diagnoses, including Alzheimer’s disease, amyotrophic lateral sclerosis, frontotemporal dementia (FTD), mild cognitive impairment, and Parkinson’s disease (PD), as well as cerebrovascular disease, using the data generated with a custom-designed neurodegenerative disease gene panel in the Ontario Neurodegenerative Disease Research Initiative (ONDRI). As expected, only ~3% of ONDRI participants harboured a monogenic variant likely driving their disease presentation. Yet, when genes were binned based on previous disease associations, we observed an enrichment of putative loss of function variants in PD genes across all ONDRI cohorts. Further, individual gene-based RVAA identified significant enrichment of rare, nonsynonymous variants in *PARK2* in the FTD cohort, and in *NOTCH3* in the PD cohort. The results indicate that there may be greater heterogeneity in the genetic factors contributing to neurodegeneration than previously appreciated. Although the mechanisms by which these genes contribute to disease presentation must be further explored, we hypothesize they may be a result of rare variants of moderate phenotypic effect contributing to overlapping pathology and clinical features observed across neurodegenerative diagnoses.

## Introduction

Neurodegenerative diseases are characterized by neuronal degeneration resulting in cognitive decline and/or motor dysfunction. Mainly manifesting in late adulthood, neurodegenerative diseases are often tightly correlated with the deposition of protein aggregates, such as beta amyloid and tau in Alzheimer’s disease (AD) and alpha-synuclein in Parkinson’s disease (PD)^1^. Although diagnoses are typically based on clinical presentation, definitive diagnosis requires post-mortem pathologic analysis to identify the pathogenic protein aggregates in situ. Further, neurodegenerative disease presentation is highly heterogeneous, and it is increasingly accepted that diagnoses exist along a spectrum, with a greater amount of mixed pathology—and overlapping clinical features—than previously thought^2^.

Genetic factors are known to increase risk of neurodegeneration and influence expression of disease features^3^; however, only ~10% of neurodegenerative disease patients are considered to have familial forms of disease, a fraction of which are caused by known rare, highly penetrant genetic variants. Similarly, while genome-wide association studies (GWASs) have identified many common GWAS-significant single-nucleotide polymorphisms (SNPs) in neurodegenerative disease cohorts, and thus have advanced the field considerably^4–6^, such variants account for only a small amount of heritable risk^7–9^. Even after considering the collective effects of both Mendelian large-effect rare mutations and common disease-associated SNPs, a considerable portion of heritability across neurodegenerative diseases remains unexplained^8,10,11^.

Recent studies have reported enrichment of rare variants in genes typically considered only in early-onset, familial neurodegenerative disease cases in cohorts with sporadic forms of disease, likely constituting a moderate effect on disease risk. For example, rare variants have been identified in patients with late-onset sporadic AD in *APP*, *PSEN1*, and *PSEN2* (ref. ^12^); in patients with both late- and early-onset sporadic PD in *SNCA*, *PARK2*, *LRRK2*, and *VPS35* (refs. ^13,14^); and in patients with both familial and sporadic amyotrophic lateral sclerosis (ALS) in *SOD1*, *FUS*, and *DNAJC7*^15^. In addition, the explanation for heterogeneity of phenotypic expression of neurodegeneration among individuals with identical rare variants is unclear, as is the potential influence of genetic factors on the overlapping clinical and pathological features of different neurodegenerative diagnoses. Such gaps in knowledge reinforce how much remains to be learned regarding genetic risk of neurodegeneration, even with respect to known neurodegenerative disease genes.

While rare variants likely account for at least a portion of the missing heritability of neurodegenerative diseases, as well as the phenotypic heterogeneity between diseases, they remain difficult to detect. Rare variants with large-effect sizes are individually very uncommon and require large samples sizes to obtain the statistical power necessary for detection—even some of the largest GWASs, with sample sizes >100,000, are still unable to detect rare disease-associated variants. However, by binning variants into gene-based groupings of their original disease associations—or by analysing each gene individually—rare variant association analyses (RVAAs) may identify gene–disease associations and explain additional disease risk even with modest sample sizes^16^.

Here we utilize targeted next-generation sequencing (NGS) data coupled with a RVAA-based binning strategy to identify the contribution of rare genetic variants in participants from the Ontario Neurodegenerative Disease Research Initiative (ONDRI) to multiple neurodegenerative disease phenotypes, including (1) AD; (2) amnestic mild cognitive impairment (MCI); (3) ALS; (4) frontotemporal dementia (FTD); and (5) PD, as well as cerebrovascular disease (CVD)^17,18^. By binning variants into disease-association-based gene groupings and individual gene-based groupings, and comparing variant enrichment to that of a cognitively normal, elderly control cohort, we seek to identify whether rare variants significantly contribute to disease presentation in ONDRI participants. Furthermore, by studying six phenotypes concurrently, we can determine whether associations exist across disease phenotypes, and whether these might account for the overlapping features often observed across neurodegenerative diseases.

## Results

### Variants likely contributing to Mendelian disease

In the total cohort of 519 ONDRI cases (Table 1), seven participants harboured nonsynonymous rare variants likely contributing to a Mendelian form of disease with each harbouring a unique variant of interest (Table 2), including one participant with AD, two participants with CVD, one participant with MCI, and three participants with PD (Supplementary Table 1). Further, seven participants carried pathogenic repeat expansions within *C9orf72* (Table 2), including four participants with ALS, two participants with FTD, and one participant with AD. Overall, monogenic variants were observed at a frequency of ~3% both before and after ancestral outlier analysis (0.027 [0.015–0.045] and 0.030 [0.016–0.052] in the total ONDRI cohort and ancestry-matched cohort, respectively). All participants were retained for subsequent analyses.

**Table 1:** Demographics of the total ONDRI cohorts and cognitively normal control cohort at baseline and the demographics of the cohorts following multivariate outlier analysis.

Cohort	Total participants			Ancestry-matched participants		
	Samples	Mean age (years ± s.d.)	Male:Female	Samples	Mean age (years ± s.d.)	Male:Female
ONDRI	519	68.6 ± 7.6	341:172	396	68.7 ± 7.9	268:128
AD	41	71.8 ± 8.0	24:17	33	71.4 ± 7.9	19:14
ALS	40	62.0 ± 8.7	24:16	32	61.9 ± 9.2	23:9
CVD	161	69.2 ± 7.4	109:50	124	69.6 ± 7.6	87:37
FTD	53	67.8 ± 7.1	34:19	39	67.5 ± 7.3	25:14
MCI	85	70.6 ± 8.3	45:40	59	71.9 ± 8.3	29:30
PD	139	67.8 ± 6.4	106:30	109	67.5 ± 6.3	85:24
Controls	189	74.0 ± 8.2	77:112	164	74.0 ± 7.9	68:96

The common variation (MAF > 0.005) captured by the ONDRISeq next-generation sequencing panel was used to perform principal component analysis on the ONDRI cases and controls accounting for variance introduced by differential ancestry and batch effects. Multivariate outlier analysis was performed using the first eight principal components. Ancestry matched refers to all samples that were not removed by the outlier analysis. Abbreviations: *AD* Alzheimer’s disease, *ALS* amyotrophic lateral sclerosis, *CVD* cerebrovascular disease, *FTD* frontotemporal dementia, *MCI* mild cognitive impairment, *ONDRI* Ontario Neurodegenerative Disease Research Initiative, *PD* Parkinson’s disease, *s.d.* standard deviation.

**Table 2:** ONDRI participants harbouring rare variants likely contributing to Mendelian forms of neurodegenerative disease and cerebrovascular disease.

Cohort	Total participants			Ancestry-matched participants		
	Samples	Monogenic rare variants	*C9orf72* expansion	Samples	Monogenic rare variants	*C9orf72* expansion
ONDRI	519	7	7	396	5	7
AD	41	1	1	33	1	1
ALS	40	0	4	32	0	4
CVD	161	2	0	124	1	0
FTD	53	0	2	39	0	2
MCI	85	1	0	59	1	0
PD	139	3	0	109	2	0

Ancestry matched refers to all samples that were not removed by the outlier analysis following principal component analysis on common variation (MAF > 0.005) captured by the ONDRISeq next-generation sequencing panel. Monogenic rare variants refers to individuals harbouring variants with a MAF < 0.01 in gnomAD v.2.1.1 (non-neuro) in a gene known to contribute to Mendelian forms of the disease of patient diagnosis and that was classified as likely pathogenic/pathogenic in ClinVar, OMIM, and/or the AlzForum mutations database. *C9orf72* expansions refers to individuals harbouring the G_4_C_2_ expansion, which were genotyped using amplicon length analysis and repeat-primed PCR; all expansions were >60 repeats in length. Abbreviations: *AD* Alzheimer’s disease, *ALS* amyotrophic lateral sclerosis, *C9orf72* chromosome 9 open reading frame 72 gene, *CVD* cerebrovascular disease, *FTD* frontotemporal dementia, *MCI* mild cognitive impairment, *ONDRI* Ontario Neurodegenerative Disease Research Initiative, *PD* Parkinson’s disease.

### Principal component analysis

Ancestry of the ONDRI cases and cognitively normal control samples were estimated by projecting their SNP loadings onto a PCA of the 1000G population (Supplementary Fig. 1). The large degree of overlap observed between the ONDRI cases, as well as the cognitively normal controls, and the European cohort of the 1000G population suggests that the participants within our study were largely of European descent. To produce a homogeneous genetic dataset, which minimizes any false discoveries due to population stratification in accordance with standard genomics quality control best practices, we performed a logistic regression of the ONDRI case and control principal components and identified the first eight components as significantly contributing to variance in the samples. Following multidimensional ancestral outlier analysis and outlier removal, the data consisted of 396 ONDRI cases and 164 cognitively normal controls (Table 1).

### Disease-association-based RVAA

**Fig. 1:**
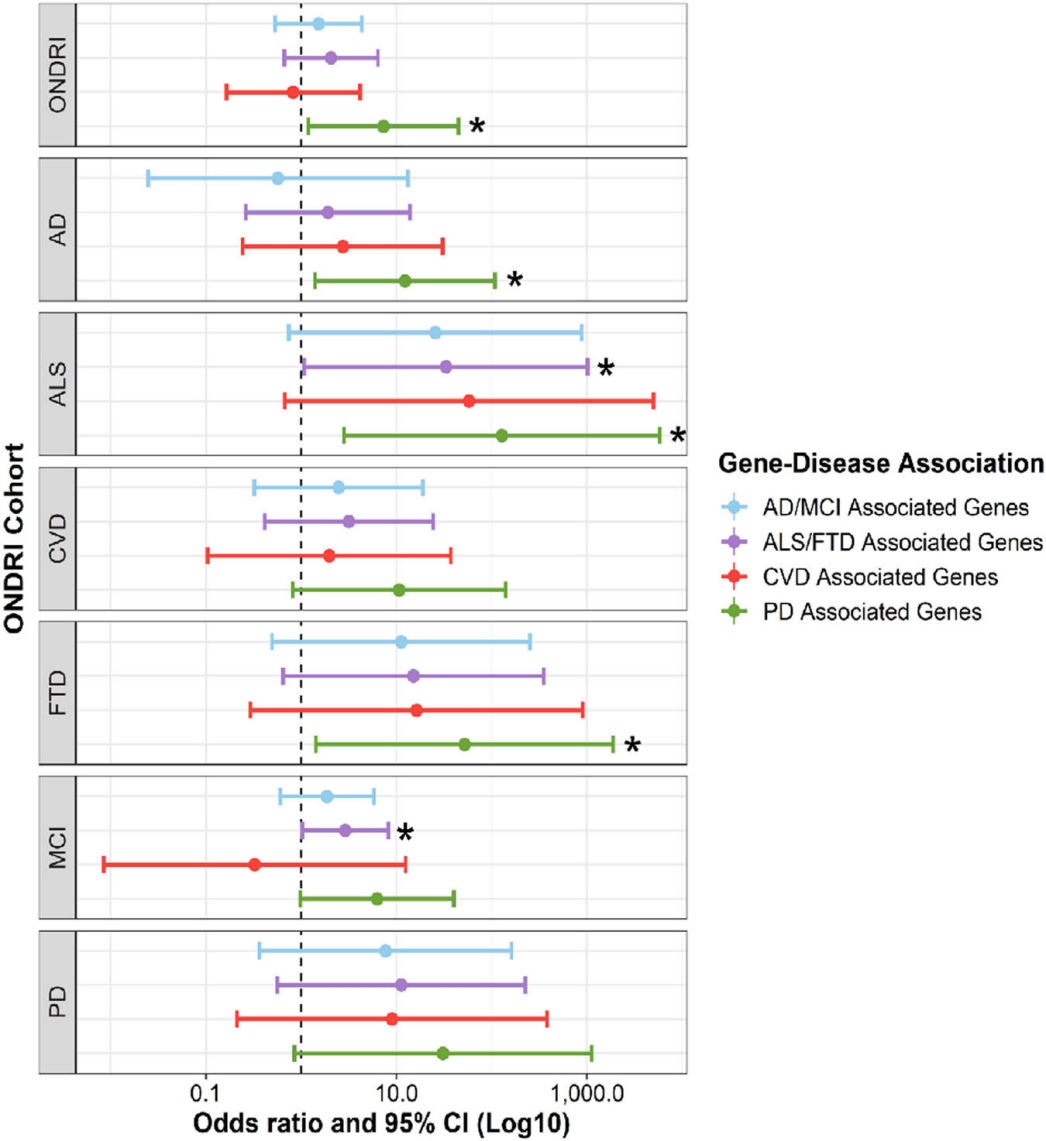
Rare variant association analysis to analyse enrichment of putative loss of function rare variants in disease-associated gene groupings. Multinomial logistic regressions adjusted for age, sex, and disease prevalence were performed to analyse enrichment of putative loss of function variants (including stop-gain, stop-loss, frameshift, splice acceptor, and splice donor sequence ontologies) identified in the 80 genes encompassed by the ONDRISeq panel, which were binned into four disease-associated gene groupings across the ONDRI cohorts compared to the control cohort. Only ancestry-matched participants were included in the analyses. The *brglm2* R package was used to fit the regression model and apply a mean bias reduction accounting for the low variant-positive counts. *p < 0.05. Abbreviations: AD Alzheimer’s disease, ALS amyotrophic lateral sclerosis, CVD cerebrovascular disease, FTD frontotemporal dementia, LOF loss of function, MCI mild cognitive impairment, ONDRI Ontario Neurodegenerative Disease Research Initiative, PD Parkinson’s disease.

All regression coefficients and standard errors obtained by the multinomial logistic regression models are summarized in Supplementary Table 2. Combined analysis of neurodegenerative disease cohorts revealed that ONDRI participants were significantly more likely to carry a putative loss of function (LOF) variant in PD-associated genes in comparison to the normal controls (OR = 7.322, *p* = 0.031; Fig. 1). Interestingly, similar significant associations were observed within each individual disease cohort when compared to controls, including for the AD (OR = 12.307, *p* = 0.023), ALS (OR = 127.744, *p* = 0.013), and FTD (OR = 51.832, *p* = 0.031) cohorts, as well as near significant results for the CVD (OR = 10.698, *p* = 0.071), MCI (OR = 6.273, *p* = 0.053), and PD (OR = 30.821, *p* = 0.061) cohorts.

In addition, ALS and MCI cases were significantly more likely to carry rare putative LOF variants in ALS/FTD-associated genes compared to the control cohort (OR = 33.169, *p* = 0.045 and OR = 2.905, *p* = 0.044, respectively; Fig. 1). The ALS cases were also more likely to carry rare putative LOF variants in AD- and CVD-associated genes, although results were not significant (OR = 25.572, *p* = 0.072 and OR = 57.857, *p* = 0.074, respectively; Fig. 1).

No differences in odds of carrying rare missense variants or possibly deleterious missense variants were observed between the participants in the neurodegenerative disease cohorts and the controls (Supplementary Fig. 2).

### Gene-based RVAA using SKAT-O

Following gene-based RVAA using the optimal unified Sequence Kernel Association Test (SKAT-O), 11 genes were identified to be likely enriched in nonsynonymous rare variants in the ONDRI cohorts compared to the controls that also had sufficient total rare variant counts for subsequent analysis. Firth logistic regression, which was used to accommodate for the limitations of SKAT-O, revealed significant differences in nonsynonymous rare variant enrichment in three genes in the ONDRI cohorts compared to the controls (Table 3), including *CHMP2B* across the combined neurodegenerative disease ONDRI cohort (OR = 0.080, *p* = 0.0008), *NEFH* in the CVD cohort (OR = 0.360, *p* = 0.036), and *PARK2* in the FTD cohort (OR = 11.602, *p* = 0.023).

**Table 3:**
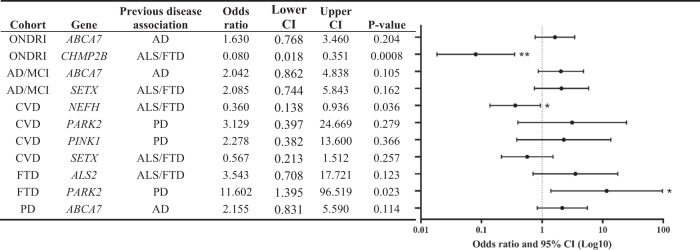
Gene-based rare variant association analyses using Firth logistic regression comparing rare variant enrichment in the ONDRI cohorts compared to the control cohort, in genes identified as having differing enrichment by SKAT-O.

Due to limitations of SKAT-O and to account for the effects of age and sex, a Firth logistic regression was performed on each gene identified by SKAT-O to compare the enrichment of rare variants in the respective ONDRI cohorts in comparison to a cognitively normal control cohort. Only ancestry-matched participants were included in the analyses. Genes that had total rare variant counts <5 or with zero rare variants in one of the cohorts were excluded from analyses. Statistical analyses were performed using the R statistical software 3.6.0 in R Studio 1.1.463. *p < 0.05; **p < 0.005; ***p < 0.0005. Abbreviations: *AD* Alzheimer’s disease, *ALS* amyotrophic lateral sclerosis, *CI* confidence interval, *CVD* cerebrovascular disease, *FTD* frontotemporal dementia, *MCI* mild cognitive impairment, *ONDRI* Ontario Neurodegenerative Disease Research Initiative, *PD* Parkinson’s disease, *SKAT-O* the optimal unified Sequence Kernel Association Test.

Similarly, SKAT-O revealed 6 genes likely enriched in nonsynonymous rare variants in the individual ONDRI disease cohorts compared to each other that also had sufficient total rare variant counts for subsequent analysis. Firth logistic regression identified two genes with a significantly different enrichment of nonsynonymous rare variants in one cohort when compared to another (Table 4), including an enrichment of variants in *NOTCH3* in the PD cohort compared to the combined AD and MCI cohort (OR = 2.986, *p* = 0.009), and an enrichment of variants in *NEFH* in the combined AD and MCI cohort compared to the CVD cohort (OR = 0.272, *p* = 0.011).

**Table 4:**
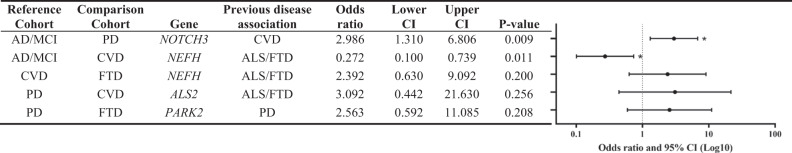
Gene-based rare variant association analyses using Firth logistic regression comparing rare variant enrichment between the individual ONDRI cohorts, in genes identified as having differing enrichment by SKAT-O.

Due to limitations of SKAT-O and to account for the effects of age and sex, a Firth logistic regression was performed on each gene identified by SKAT-O to compare the enrichment of rare variants between the individual ONDRI disease cohorts. Only ancestry-matched participants were included in the analyses. Genes that had total rare variant counts <5, or with zero rare variants in one of the cohorts were excluded from analyses. Statistical analyses were performed using the R statistical software 3.6.0 in R Studio 1.1.463. *p < 0.05; **p < 0.005; ***p < 0.0005. Abbreviations: *AD* Alzheimer’s disease, *ALS* amyotrophic lateral sclerosis, *CI* confidence interval, *CVD* cerebrovascular disease, *FTD* frontotemporal dementia, *MCI* mild cognitive impairment, *ONDRI* Ontario Neurodegenerative Disease Research Initiative, *PD* Parkinson’s disease, *SKAT-O* the optimal unified Sequence Kernel Association Test.

## Discussion

As previously described, a large amount of missing heritability remains across neurodegenerative diagnoses and little is known regarding the contribution of rare genetic factors to the heterogeneous presentation of these diseases. Due to the established infrequency of Mendelian forms of neurodegenerative phenotypes^19–22^, it was not surprising that few ONDRI participants harboured monogenic variants likely driving their disease presentation, including seven carriers of likely monogenic nonsynonymous, rare variants—defined as variants previously reported as pathogenic within relevant mutations databases and the literature in respect to the participant’s diagnosis—and seven carriers of the pathogenic *C9orf72* repeat expansion. Yet, the low frequency of monogenic variants observed in our cohorts has highlighted the need to investigate the contribution of rare variants in genes previously associated with neurodegeneration to the presentation of the entire spectrum of neurodegenerative and CVD diagnoses utilizing RVAA. Associations between specific neurodegenerative diagnoses and known neurodegeneration genes were identified with SKAT-O and subsequent logistic regression, as well as with disease-association-based RVAA. Our principal findings included associations between (1) nonsynonymous rare variants in *PARK2* and the FTD cohort; (2) nonsynonymous rare variants in *NOTCH3* and the PD cohort; (3) rare, putative LOF variants in PD-associated genes across the entire ONDRI cohort; and (4) rare, putative LOF variants in ALS/FTD-associated genes in the ALS and MCI cohorts.

Nonsynonymous rare variants in *PARK2* were enriched in the FTD cohort. While *PARK2* is well-established to be associated with autosomal recessive familial PD^23^ and potentially with autosomal dominant sporadic PD^24,25^, it has not been previously associated with FTD. However, both PD and FTD are influenced by lysosome dysfunction, which can be exacerbated by mutated *PARK2*^26^. The variants identified in the FTD cohort were all of heterozygous zygosity, and two had been previously reported as variants of uncertain significance in ClinVar (i.e. p.Arg402Cys and p.Pro437Leu). Although the variants may have contributed to the FTD diagnoses in our cohort, it remains possible that the variants had a moderate phenotypic effect and/or decreased penetrance. If so, our result may be consistent with some of the heterogeneity and overlap often seen across neurodegenerative disease presentations, therefore highlighting the potential impact of unexpected rare variation to features of disease, which is an area of neurogenetics that must be further explored.

One example of how rare variants may contribute to intermediate phenotypes of neurodegeneration, rather than a diagnosis itself, is demonstrated by rare variants in *NOTCH3* among participants with PD. Typically, rare variants within *NOTCH3* are associated with a monogenic subtype of CVD called cerebral autosomal dominant arteriopathy with subcortical infarcts and leukoencephalopathy (CADASIL). Leukoencephalopathy associated with CADASIL can be manifested as white matter hyperintensities seen on T2-weighted magnetic resonance imaging scans. We previously observed that PD participants harbouring rare *NOTCH3* variants had double the volume of white matter hyperintensities than those that did not^27^. Herein, we did not observe an association between PD and *NOTCH3* when compared to the controls, but the history of CVD in the control cohort was unknown. An association was observed between PD and *NOTCH3* when compared to the combined AD and MCI cohort, which was of particular interest as ONDRI excluded participants from the AD and MCI cohorts who had significant evidence of vascular pathology^18^. Therefore, this result seemingly supports the hypothesis that rare *NOTCH3* variation may be contributing to cerebrovascular features within patients with PD^27^.

The gene-based RVAA also identified *CHMP2B* as having significantly fewer variants in the entire ONDRI cohort compared to controls, and *NEFH* as having significantly fewer variants in the CVD cohort compared to the controls or the combined AD/MCI cohort. These results could be interpreted as protection against neurodegenerative diseases and cerebrovascular phenotypes from rare variants in *CHMP2B* and *NEFH*, respectively. However, the association within *CHMP2B* was likely driven by a single splicing variant (c.34 + 8C > T) harboured by the only three ONDRI participants with rare *CHMP2B* variants and five of the eight controls with rare *CHMP2B* variants. The variant had a MAF in ExAC of 2.80E−3 and was previously reported in ClinVar as benign. It is possible that the variants in *CHMP2B* and *NEFH* may have gain-of-function protective effects, explaining the unexpected signal; however, functional assays are needed for confirmation. Based on the large amount of influence from single variants in these potentially protective results, specifically in the case of *CHMP2B*, and no previously established protective effects for either gene within the literature, further interpretation remains unclear.

No ‘expected’ rare variant associations were observed between the individual neurodegenerative disease cohorts and genes previously associated with the disease cohorts in the gene-based analysis. For example, there were no associations between rare variants in *APP*, *PSEN1*, or *PSEN2* with the combined AD and MCI cohort. Although this was unsurprising due to the low frequency of monogenic variants we identified in the ONDRI cohorts (Table 2), it suggests there may be other genetic determinants driving disease presentation and progression. So, to maximize analytic power, we also assessed rare variant frequency in groups of genes, based on the disease with which the genes have been most commonly associated. Across all neurodegenerative diagnoses, rare, putative LOF variants in PD-associated genes were enriched when compared to the control cohort. Although unsurprising in the PD cohort, this interesting trend was observed in all five remaining neurodegenerative disease cohorts individually, as well as in the combined ONDRI cohort.

When we examined the individual genes that contributed to the association, 8 of the 13 putative LOF PD-associated variant-positive participants (61.5%) harboured variants in *MC1R*. Specifically, the putative LOF variants in *MC1R* were identified in the CVD, FTD, MCI, and PD cohorts, as well as in one control participant. *MC1R* on chromosome 16 encodes a receptor typically involved in the regulation of melanin pigment within the skin, but is also expressed in the periaqueductal grey matter of the brain^28^. The gene was originally associated with PD in a study by Tell-Marti et al.^29^, in which a common missense variant (p.Arg160Trp) was associated with the disease in a Spanish population. Previous research has also suggested an association between both red hair and melanoma—for which *MC1R* variants are a risk factor—and PD^30,31^, and the MC1R protein was found to be neuroprotective in dopaminergic neurons, which are integral to PD pathology. Unfortunately, the association between *MC1R* and PD is controversial, with multiple studies unable to replicate the finding^32,33^, and to date, no strong evidence linking *MC1R* variation to any other neurodegenerative disease exists.

We also observed a significant enrichment of rare, putative LOF variants in ALS/FTD-associated genes in the ALS and MCI cohorts. No single gene stood out in the analysis and it is important to recognize that the number of participants in each cohort harbouring rare putative LOF variants was low, so we are cautious to not draw conclusions from these results given the small sample sizes. However, it cannot be discounted that the enrichment signal within the MCI cohort may suggest the participants’ potential to progress to FTD, rather than AD. Typically, we anticipate that amnestic MCI patients will progress to an AD phenotype or will not progress at all, yet the possibility remains that presentation will develop into a variant of FTD and follow-up of the MCI participants in ONDRI remains imperative.

Our study did have limitations that deserve comment. The analysis was largely limited by modest sample sizes, particularly after accounting for variance resulting from differential ancestry and batch effects. Combined with the inherent rarity of the variants, the number of variant-positive participants in each cohort remained small. Yet, the study still identified interesting signals that are reasonable contenders for replication within larger cohorts and hypothesis generating for further analyses. Further, apart from basic demographic information and Montreal Cognitive Assessment scores to define the control cohort as cognitively normal upon enrolment, no further data were available. Therefore, it is unclear whether control participants had any history of CVD without cognitive impairment and analyses may not have been sensitive to signals from the CVD-associated genes on the ONDRISeq panel, as highlighted by the association between *NOTCH3* and PD when compared to the AD/MCI cohort, but not the controls. Our analyses were also limited to individuals of probable European ancestry and replication in other populations is necessary. Finally, our results were restricted to the 80 genes covered by our custom targeted NGS panel^34^ and identification of novel genes associated with neurodegenerative disease was not possible. Despite this, we still identified associations between specific neurodegenerative diagnoses and known neurodegeneration genes, such as the enrichment of *PARK2* variants in FTD.

Our analyses allowed us to observe considerable heterogeneity in the genetic contribution underlying neurodegenerative disease presentation. While we could not conclude that the rare variants observed were driving diagnoses in all instances, it is reasonable to assume that some of the variability observed in neurodegenerative disease presentation may be driven by the rare variants observed in ‘atypical’ neurodegenerative disease genes. Future analyses are required to replicate our findings; however, our study demonstrates the potential for RVAA as an approach to identify genes in which rare variants may have moderate and somewhat unanticipated phenotypic effects in certain neurodegenerative disease cohorts, either by directly influencing disease pathology or by potentially contributing to the overlapping features across neurodegenerative disease. Overall, this may suggest a more complex genetic architecture of neurodegeneration than the familiar simple monogenic model of inheritance in which a variant fully explains a clinical phenotype.

## Methods

### Sample collection, ethics approval, and DNA sequencing

In total, 520 individuals passed ONDRI preliminary screening^17^. Of those, 519 participants had a blood sample collected, from which genomic DNA was extracted. Study ethics approval was obtained from the Research Ethics Boards at Baycrest Centre for Geriatric Care (Toronto, Ontario, Canada); Centre for Addiction and Mental Health (Toronto, Ontario, Canada); Elizabeth Bruyère Hospital (Ottawa, Ontario, Canada); Hamilton General Hospital (Hamilton, Ontario, Canada); McMaster (Hamilton, Ontario, Canada); London Health Sciences Centre (London, Ontario, Canada); Parkwood Hospital (London, Ontario, Canada); St Michael’s Hospital (Toronto, Ontario, Canada); Sunnybrook Health Sciences Centre (Toronto, Ontario, Canada); The Ottawa Hospital (Ottawa, Ontario, Canada); and University Health Network-Toronto Western Hospital (Toronto, Ontario, Canada). All participants provided written, informed consent in accordance with the Research Ethics Boards and regulatory requirements. DNA was also obtained from 189 cognitively normal control genomic DNA samples obtained from the GenADA study^35^.

All DNA samples were sequenced using the targeted NGS panel, ONDRISeq, on the Illumina MiSeq Personal Genome Sequencer (Illumina, San Diego, CA, United States) and raw sequencing data were processed with a custom bioinformatics workflow. Briefly, FASTQ files were imported into CLC Bio Genomics Workbench v10 (CLC Bio, Aarhus, Denmark) to perform pre-processing and variant annotation, which produced a variant calling format (VCF) file and binary alignment map (BAM) file for each participant. Detailed methodology outlining DNA isolation, DNA sequencing, and sequencing analysis has been previously described^36^.

### Identification of likely Mendelian variants

ONDRISeq VCF files of the ONDRI cases were imported into VarSeq® (Golden Helix, Bozeman, MT, United States) and variants were annotated with sequence ontologies. Minor allele frequencies (MAFs) were obtained from the Genome Aggregation Database (gnomAD v.2.0.1v3 non-neuro)^37^. Rare (MAF < 0.01), nonsynonymous variants were prioritized. Further assessment of variants was performed to identify those in genes known to contribute to Mendelian forms of the patient’s disease of diagnosis and those classified as pathogenic or likely pathogenic in ClinVar^38^, Online Mendelian Inheritance in Man (OMIM)^39^, and/or the Alzforum Mutation Database^40^. All identified variants were considered those likely to be contributing to Mendelian forms of disease.

All samples were genotyped for *C9orf72* using both amplicon length analysis and repeat-primed polymerase chain reaction (PCR), as previously described^41^. Harbouring >30 repeats is a commonly accepted genetic cause of ALS and FTD^41,42^, and therefore was the cutoff used to determine those with pathogenic repeat expansions.

### Ancestry matching and estimation

The ONDRISeq VCF files of all cases and controls were merged and filtered to include only SNPs within exonic and splicing regions with a MAF > 0.005 in the Genome Aggregation Database (gnomAD v.2.0.1) using VarSeq®. Variants that were located on the sex chromosomes or within the *MAPT* gene were excluded, due to potential influence from the cohort’s sex distribution and a common haplotype variation found across the gene, respectively. The filtered merged VCF was processed with a bash-based tool that contains a collection of scripts necessary to run region-based RVAA, 'Exautomate'^43^, to produce PLINK compatible MAP and PED files. SNP & Variation Suite v8.8.3 (SVS; Golden Helix Inc.) was used to perform linkage disequilibrium (LD) pruning (threshold = 0.5) and a principal component analysis (PCA) was performed to identify the genetic ancestry.

In accordance with standard quality control in genomic studies, a logistic regression analysis was performed within R on the generated principal components. To identify individuals with divergent ancestries to minimize false discoveries due to population stratification, a multidimensional outlier analysis (multiplier = 1.5) was performed within SVS using the significant components to identify outlier samples based on ancestral variation and batch effects, which were not included in the RVAAs described below.

To predict the genomic ancestry of the samples, we used the whole-genome sequences from the 1000 Genomes Project (1000G; *N* = 2693), which are binned into ancestral groups, including African, Admixed American, East Asian, European, and South Asian^44^. The 1000G VCFs were merged and filtered to include only SNPs within the exonic and splicing regions captured by the ONDRISeq panel with an MAF > 0.005 in gnomAD. The resulting filtered merged VCF was processed using 'Exautomate' to produce MAP and PED files and a PCA was performed using the SNPRelate Bioconductor R package (v1.22.0; LD pruning threshold = 0.5)^45^. The SNP loadings from this PCA and the PED file of the ONDRI cases and controls were used to project the ONDRI cases and controls onto the components of the 1000G PCA^46^.

### Rare variant association analysis

The VCF files of all ancestry-matched ONDRI cases and controls were imported into the variant annotation software, VarSeq®. Variants were annotated with sequence ontologies, MAFs from Exome Aggregation Consortium (ExAC v1.0), and in silico prediction scores from Combined Annotation Dependent Depletion (CADD; v1.3)^47^, Sorting Intolerant from Tolerant (SIFT)^48^, and PolyPhen-2^49^. Variants were prioritized and variants with a sequence ontology of stop-gain, stop-loss, splicing acceptor, splicing donor, frameshift, or missense, and a MAF < 0.01 in ExAC were included in subsequent analyses. Both heterozygous and homozygous variants were retained for RVAAs. Variants were binned into three groups: (1) putative LOF variants (including stop-gain, stop-loss, frameshift, splice acceptor, and splice donor sequence ontologies); (2) missense variants; and (3) possibly deleterious missense variants (including missense variants with either a CADD Phred ≥20 or a likely damaging/damaging prediction from both SIFT and PolyPhen-2). Carriers of these variants were considered ‘variant-positive’ and ‘variant negative’, respectively.

**Fig. 2:**
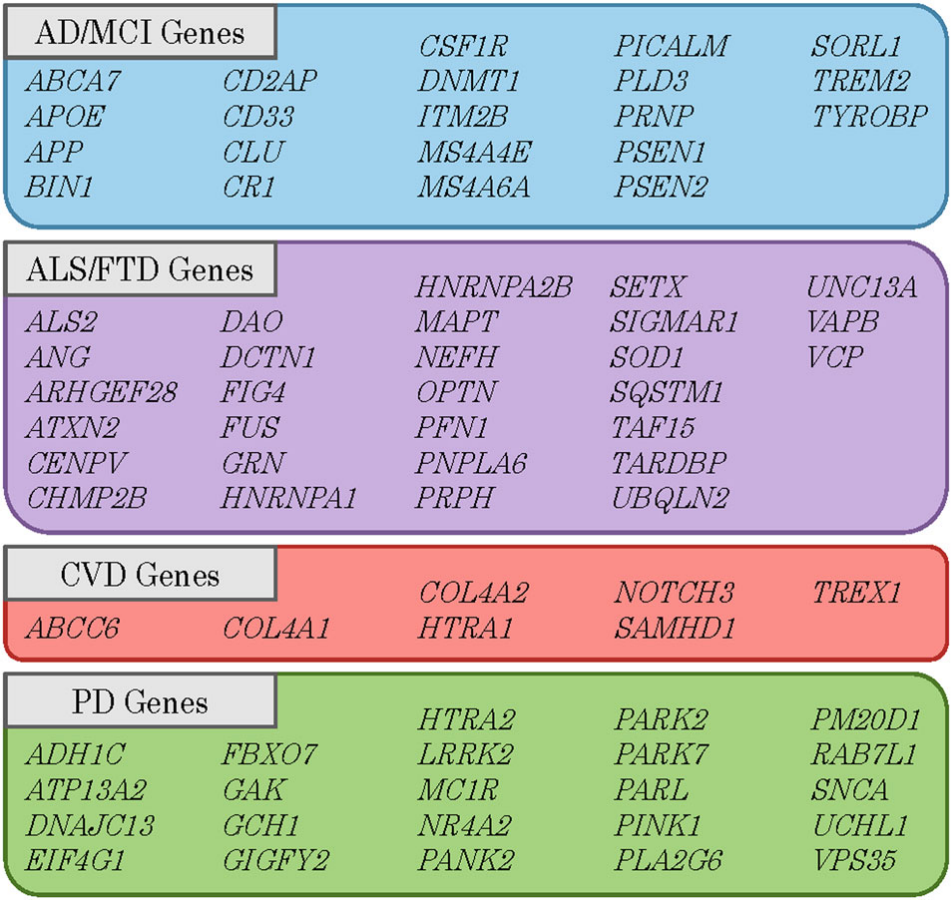
Binning of genes included on the ONDRISeq next-generation sequencing panel. Genes were binned based on the most well-established previous disease association, as determined by Farhan et al.^34^, for use in the rare variant association analyses.

Variants were also binned into groups based on the previous disease association of the gene in which the variant was located. In total, the 80 genes encompassed by the ONDRISeq panel were binned into four disease-association groups: (1) AD/MCI-associated genes; (2) ALS/FTD-associated genes; (3) PD-associated genes; and (4) CVD-associated genes based on the most well-established previous disease association, as determined by Farhan et al. (Fig. 2)^34^.

RVAAs were performed using multinomial logistic regression models. A model was produced for each variant subgroup (putative LOF, missense, and possibly deleterious missense) to compare the number of variant-positive individuals in each of the ONDRI disease cohorts to the cognitively normal control cohort, while correcting for age and sex. In addition, participants were weighted to better reflect disease prevalence in the general elderly population, accounting for potential inference bias as a result of the non-probability sampling mechanism^50–55^. The *brglm2* R package (v0.6.2)^56^ was used to fit the regression models and apply a mean bias reduction^57^ that account for the low variant-positive counts.

A gene-based RVAA, SKAT-O, was also performed using the script package 'Exautomate'^43^. This method identified specific genes covered by ONDRISeq with an increased frequency of nonsynonymous, rare variants (MAF < 0.01, ExAC) in the disease cohorts compared to controls, and in the disease cohorts compared to each other. To maximize sample sizes, the AD and amnestic MCI cohorts were combined for SKAT-O analyses. As SKAT-O was not able to account for multi-nucleotide variants, follow-up analyses were performed on SKAT-O results with a detected signal using Firth logistic regression, adjusting for age and sex, using the *brglm2* R package. Genes that had total rare variant counts between the two cohorts of <5 or with zero rare variants in one of the cohorts were excluded from analyses.

Analyses were performed using R statistical software 3.6.0^58^ in R Studio 1.1.463 and data visualization was performed using the *ggplot2* R package (v3.3.s)^59^. Significance for all regression analyses was measured at an alpha-level of *p* < 0.050, although regression results with *p* < 0.075 were still reported.

### Reporting summary

Further information on research design is available in the Nature Research Reporting Summary linked to this article.

## Supplementary information

Supplementary Information

Reporting Summary

## Data Availability

In accordance with the Ontario Neurodegenerative Disease Research Initiative (ONDRI) with the Ontario Brain Institute, all baseline data from ONDRI are available upon request at https://www.braincode.ca/. All data have been de-identified. To gain access to the data, an account request must be made to help@braincode.ca. This process requires the applicant to provide their contact information and association, which are then verified. Data access will require the completion of a Data Access Request, which will be provided following the initial account request. Further details regarding data access can be found at https://www.braincode.ca/content/getting-started. The data are not available publicly outside of the Brain-CODE portal due to information that could compromise the privacy of the research participants.

## Code availability

Next-generation sequencing data were processed using a bioinformatics workflow in CLC Bio Genomics Workbench v10 (CLC Bio, Aarhus, Denmark) and annotated using VarSeq® (Golden Helix, Bozeman, MT, United States). The Optimal Sequence Kernal Association Test was performed using the Exhautomate package, available at https://github.com/exautomate/Exautomate-Core. Remaining statistical analyses were performed using R statistical software 3.6.0 in R Studio 1.1.463 and data visualization was performed using the ggplot2 R package (v3.3.s).
