# Supplementary material for: Contribution of rare variant associations to neurodegenerative disease presentation
Source: NPJ Genom Med. 2021 Sep 28;6:80. doi: 10.1038/s41525-021-00243-3 (PMC8478934; doi:10.1038/s41525-021-00243-3)
Supplement: Supplementary file 1 — Supplementary Information [file 41525_2021_243_MOESM1_ESM.pdf]

Contribution of rare variant associations to neurodegenerative disease presentation

Supplementary Tables

Supplementary Table 1. Nonsynonymous rare variants likely contributing to Mendelian forms of neurodegenerative disease and cerebrovascular disease identified in the total ONDRI cohort.

Gene	cDNA alteration	Protein alteration	Reference SNP identifier	Sequence ontology	MAF (gnomAD)	CADD Phred	Previous disease association	Participants harbouring variant (cohort)
APP	c.2137G>A	p.Ala713Thr	rs63750066	Missense	2.88e-5	34.0	AD[1-3]	1 (AD)
GCH1	c.671A>G	p.Lys224Arg	rs41298442	Missense	4.70e-5	13.9	PD[4-6]	1 (PD)
LRRK2	c.6055G>A	p.Gly2019Ser	rs34637584	Missense	2.26e-4	35	PD[7,8]	1 (PD)
NOTCH3	c.544C>T	p.Arg182Cys	rs28933697	Missense	4.70e-5	31.0	CADASIL[9,10]	1 (CVD)
NOTCH3	c.580T>C	p.Cys194Arg	rs1568361818	Missense	NA	23.5	CADASIL[11]	1 (CVD)
PSEN1	c.118_120delGAC	p.Asp40del	rs759538127	In-frame deletion	1.40e-4	14.1	AD[12]	1 (MCI)
SNCA	c.150T>G	p.His50Gln	rs201106962	Missense	8.28e-5	4.9	PD[13]	1 (PD)

Nonsynonymous rare variants likely contributing to Mendelian forms of neurodegenerative disease were considered variants with a MAF < 0.01 in gnomAD v.2.1.1 (non-neuro) in a gene known to contribute to Mendelian forms of the disease of patient diagnosis and that was classified as likely pathogenic/pathogenic in ClinVar, OMIM, and/or the AlzForum mutations database. Abbreviations: AD, Alzheimer's disease; *APP*, amyloid precursor protein gene; CADASIL, cerebral autosomal dominant arteriopathy with subcortical infarcts and leukoencephalopathy; CADD Phred, combined annotation dependent depletion Phred; cDNA, coding DNA; *COL4A2*, collagen type IV alpha 2 chain protein; CVD, cerebrovascular disease; *GCH1*, GTP cyclohydrolase 1 gene; *LRRK2*, leucine rich repeat kinase 2 gene; MAF, minor allele frequency; MCI, mild cognitive impairment; NA, not applicable; *NOTCH3*, notch receptor 3 gene; PD, Parkinson's disease; *PSEN1*, presenilin 1 gene; *SNCA*, synuclein alpha gene; SNP, single nucleotide polymorphism.

Supplementary Table 2. Regression coefficients (standard error) of the multinomial logistic regressions used for rare variant association analysis comparing the enrichment of rare variants in four disease-associated gene groupings in the ONDRI cohorts compared to cognitively normal controls.

Cohort	AD/MCI Associated Genes	ALS/FTD Associated Genes	CVD Associated Genes	PD Associated Genes
Putative loss of function variants				
**ONDRI**	0.423 (0.534)	0.725 (0.576)	-0.194 (0.822)	1.991 (0.924)*
**AD**	-0.560 (1.601)	0.646 (1.014)	1.007 (1.233)	2.510 (1.107)*
**ALS**	3.242 (1.805)	3.502 (1.747)*	4.058 (2.272)	4.850 (1.947)*
**CVD**	0.904 (1.039)	1.156 (1.039)	0.680 (1.499)	2.370 (1.313)
**FTD**	2.415 (1.591)	2.713 (1.607)	2.790 (2.049)	3.948 (1.833)*
**MCI**	0.626 (0.575)	1.066 (0.529)*	-1.125 (1.862)	1.836 (0.950)
**PD**	2.039 (1.555)	2.420 (1.531)	2.196 (1.910)	3.428 (1.832)
Missense variants				
**ONDRI**	0.047 (0.206)	-0.183 (0.205)	-0.116 (0.217)	-0.051 (0.208)
**AD**	0.240 (0.384)	-0.239 (0.386)	-0.355 (0.425)	-0.350 (0.384)
**ALS**	0.291 (1.113)	-0.048 (1.115)	0.441 (1.125)	-0.383 (1.119)
**CVD**	-0.088 (0.442)	-0.384 (0.440)	-0.248 (0.477)	0.047 (0.443)
**FTD**	0.065 (0.986)	0.045 (0.987)	0.258 (1.013)	-0.109 (1.001)
**MCI**	0.107 (0.228)	-0.056 (0.228)	-0.028 (0.237)	-0.065 (0.230)
**PD**	0.242 (0.873)	-0.339 (0.876)	0.229 (0.909)	-0.083 (0.885)
Possibly deleterious missense variants				
**ONDRI**	-0.209 (0.235)	-0.056 (0.216)	-0.141 (0.236)	0.123 (0.210)
**AD**	0.146 (0.420)	0.275 (0.391)	-0.709 (0.512)	-0.526 (0.439)
**ALS**	1.010 (1.134)	0.345 (1.145)	0.615 (1.158)	0.324 (1.134)
**CVD**	-0.222 (0.513)	-0.306 (0.480)	-0.189 (0.523)	0.302 (0.444)
**FTD**	0.478 (1.024)	0.299 (1.009)	0.263 (1.069)	0.670 (1.003)
**MCI**	-0.109 (0.262)	0.133 (0.237)	-0.137 (0.261)	0.381 (0.230)
**PD**	0.171 (0.943)	0.043 (0.905)	0.489 (0.934)	0.178 (0.907)

Multinomial logistic regressions adjusted for age, sex, and disease prevalence were performed to analyze enrichment of rare variants identified in the 80 genes encompassed by the ONDRISeq panel. The brglm2 R package was used to fit the regression model and apply a mean bias reduction accounting for the low variant positive counts. *p-value < 0.05. Abbreviations: AD, Alzheimer's disease; ALS, amyotrophic lateral sclerosis; CVD, cerebrovascular disease; FTD, frontotemporal dementia; MCI mild cognitive impairment; ONDRI, Ontario Neurodegenerative Disease Research Initiative; PD, Parkinson's disease.

Supplementary Figures

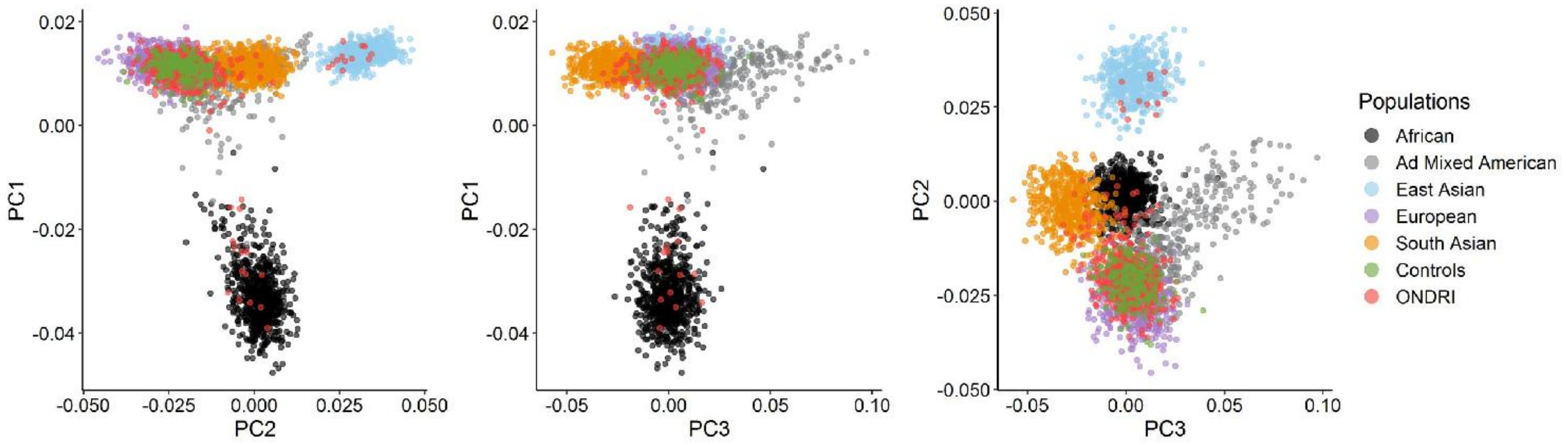

Supplementary Figure 1. Ancestry estimate of the ONDRI cases and cognitively normal control cohort using a principal component analysis (PCA) of data from the 1000 Genomes Project. Whole genome sequencing VCFs from 1000G were merged and filtered to include SNPs (MAF > 0.005, gnomAD) within the exonic and splicing regions captured by the ONDRISeq panel. A PCA was run using the *SNPRelate* Bioconductor R package (LD pruning threshold = 0.5). SNP loadings from the PCA were used to project the ONDRI cases and controls onto the components to estimate the participant's ancestries. Abbreviations: ONDRI, Ontario Neurodegenerative Disease Research Initiative; PC, principal component.

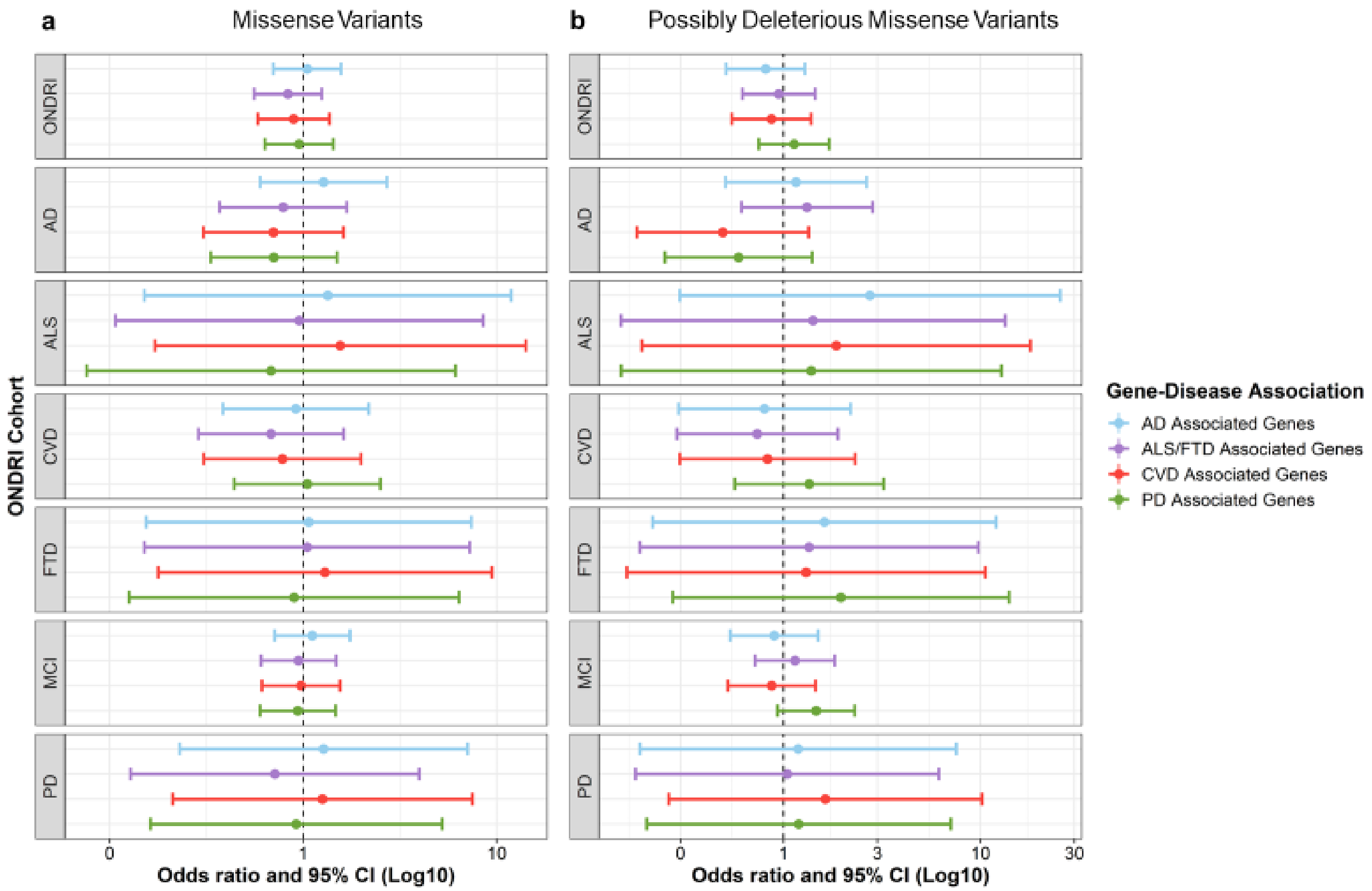

Supplementary Figure 2. Rare variant association analysis to analyze enrichment of rare missense variants in disease-associated gene groupings. Multinomial logistic regressions adjusted for age, sex, and disease prevalence were performed to analyze enrichment of (a) missense variants, and (b) possibly deleterious missense variants identified in the 80 genes encompassed by the ONDRISeq panel, which were binned into four disease-associated gene groupings across the ONDRI cohorts compared to the control cohort. Only ancestry matched participants were included in the analyses. The brglm2 R package was used to fit the regression model and apply a mean bias reduction accounting for the low variant positive counts. Abbreviations: AD, Alzheimer's disease; ALS, amyotrophic lateral sclerosis; CVD, cerebrovascular disease; FTD, frontotemporal dementia; MCI mild cognitive impairment; ONDRI, Ontario Neurodegenerative Disease Research Initiative; PD, Parkinson's disease.

References

1 Carter, D. A. *et al.* More missense in amyloid gene. *Nat Genet* **2**, 255-256, doi:10.1038/ng1292-255 (1992).
2 Armstrong, J., Boada, M., Rey, M. J., Vidal, N. & Ferrer, I. Familial Alzheimer disease associated with A713T mutation in APP. *Neurosci Lett* **370**, 241-243, doi:10.1016/j.neulet.2004.08.026 (2004).
3 Pera, M. *et al.* Distinct patterns of APP processing in the CNS in autosomal-dominant and sporadic Alzheimer disease. *Acta Neuropathol* **125**, 201-213, doi:10.1007/s00401-012-1062-9 (2013).
4 Leuzzi, V. *et al.* Autosomal dominant GTP-CH deficiency presenting as a dopa-responsive myoclonus-dystonia syndrome. *Neurology* **59**, 1241-1243, doi:10.1212/wnl.59.8.1241 (2002).
5 Saunders-Pullman, R. *et al.* Phenylalanine loading as a diagnostic test for DRD: interpreting the utility of the test. *Mol Genet Metab* **83**, 207-212, doi:10.1016/j.ymgme.2004.07.010 (2004).
6 Trender-Gerhard, I. *et al.* Autosomal-dominant GTPCH1-deficient DRD: clinical characteristics and long-term outcome of 34 patients. *J Neurol Neurosurg Psychiatry* **80**, 839-845, doi:10.1136/jnnp.2008.155861 (2009).
7 Zabetian, C. P. *et al.* LRRK2 G2019S in families with Parkinson disease who originated from Europe and the Middle East: evidence of two distinct founding events beginning two millennia ago. *Am J Hum Genet* **79**, 752-758, doi:10.1086/508025 (2006).
8 Di Fonzo, A. *et al.* A frequent LRRK2 gene mutation associated with autosomal dominant Parkinson's disease. *Lancet* **365**, 412-415, doi:10.1016/S0140-6736(05)17829-5 (2005).
9 Joutel, A. *et al.* Strong clustering and stereotyped nature of Notch3 mutations in CADASIL patients. *Lancet* **350**, 1511-1515, doi:10.1016/S0140-6736(97)08083-5 (1997).
10 Joutel, A. *et al.* Skin biopsy immunostaining with a Notch3 monoclonal antibody for CADASIL diagnosis. *Lancet* **358**, 2049-2051, doi:10.1016/S0140-6736(01)07142-2 (2001).
11 Kalimo, H., Ruchoux, M. M., Viitanen, M. & Kalaria, R. N. CADASIL: a common form of hereditary arteriopathy causing brain infarcts and dementia. *Brain Pathol* **12**, 371-384, doi:10.1111/j.1750-3639.2002.tb00451.x (2002).
12 Nygaard, H. B., Lippa, C. F., Mehdi, D. & Baehring, J. M. A Novel Presenilin 1 Mutation in Early-Onset Alzheimer's Disease With Prominent Frontal Features. *Am J Alzheimers Dis Other Demen* **29**, 433-435, doi:10.1177/1533317513518653 (2014).
13 Khalaf, O. *et al.* The H50Q mutation enhances alpha-synuclein aggregation, secretion, and toxicity. *J Biol Chem* **289**, 21856-21876, doi:10.1074/jbc.M114.553297 (2014).

The ONDRI Investigators:

Sabrina Adamo[37], Stephen Arnott[4], Rob Bartha[1,38], Derek Beaton[39], Courtney Berezuk[37], Alanna Black[40], Alisia Bonnick[6], David Breen[41-43], Don Brien[44], Susan Bronskill[45], Dennis Bulman[46], Ying Chen[44], Marvin Chum[30], Brian Coe[44], Ben Cornish[47], Sherif Defrawy[48], Jane Lawrence Dewar[20], Roger A. Dixon[49], Frederico Faria[40], Julia Fraser[47], Mahdi Ghani[29], Barry Greenberg[50], Hassan Haddad[51], Wendy Hatch[48], Melissa Holmes[37], Chris Hudson[52], Peter Kleinstiver[10], Elena Leontieva[52], Brian Levine[4], Wendy Lou[7], Efrem Mandelcorn[48], Ed Margolin[48], Connie Marras[25], William McIlroy[47], Paula McLaughlin[53], Manuel Montero Odasso[40], Doug Munoz[44], David Munoz[54], Nuwan Nanayakkara[1,10], JB Orange[55], Miracle Ozzoude[37], Alicia Peltsch[56], Joel Ramirez[37], Natalie Rashkovan[11], Angela C. Roberts[55,57], Yanina Sarquis Adamson[40], Christopher J.M. Scott[6], Michael Strong[10,58], Stephen Strothers[4,59], Sujeevini Sujanthan[60], Sean Symons[6,61], Faryan Tayyari[52], Athena Theyers[4], Angela Troyer[62], Karen Van Ooteghem[47], John Woulfe[13,14], Mojdeh Zamyadi[59], Guangyong Zou[10]

37. *Hurvitz Brain Sciences Research Program Sunnybrook Health Sciences Research Program, Sunnybrook Health Sciences Centre, Toronto, ON;*
38. *Department of Medical Biophysics, Schulich School of Medicine and Dentistry, Western University, London, ON;*
39. *Data Science & Advanced Analytics, Unity Health Toronto, Toronto, ON;*
40. *Gait and Brain Lab, Department of Medicine (Geriatrics) and Epidemiology and Biostatistics, Schulich School of Medicine and Dentistry, Western University, London, ON;*
41. *Centre for Clinical Brain Sciences, University of Edinburgh, Edinburgh, United Kingdom;*
42. *Anne Rowling Regenerative Neurology Clinic, University of Edinburgh, Edinburgh, United Kingdom;*
43. *Usher Institute of Population Health Sciences and Informatics, University of Edinburgh, Edinburgh, United Kingdom*
44. *Centre for Neuroscience Studies, Queen's University, Kingston, ON;*
45. *ICES, Toronto, ON, Canada; Instutite of Health Policy, Managemen & Evaluation, Dalla Lana School of Public Health, University of Toronto, Toronto, ON, Canada; Sunnybrook Research Institute, Toronto, ON;*
46. *Department of Medical Genetics, University of Alberta, Edmonton, AB;*
47. *Department of Kinesiology, University of Waterloo, Waterloo, ON;*
48. *Department of Ophtalmology and Vision Sciences, University of Toronto, Toronto, ON;*
49. *Department of Psychology (Science), Neuroscience and Mental Health Institute, University of Alberta, Edmonton, AB;*
50. *Toronto Dementia Research Alliance, University Health Network, Toronto, ON;*
51. *Centre for Functional and Metabolic Mapping, Robarts Research Institute, University of Western Ontario, London, ON;*
52. *School of Optometry and Vision Science, University of Waterloo, Waterloo, ON;*
53. *Nova Scotia Health, Halifax, NS; Department of Medicine (Geriatrics), Dalhousie University, Halifax, NS*
54. *Department of Laboratory Medicine, St Michael's Hospital, Unity Health, Toronto, ON;*

[55.] *School of Communication Sciences and Disorders, Faculty of Health Sciences; Canadian Centre for Activity and Aging, Western University, London, ON;*
[56.] *Faculty of Engineering and Applied Science, Queen's University, Kingston, ON;*
[57.] *Roxelyn and Richard Pepper Department of Communication Sciences and Disorders, Northwestern University, Evanston, IL;*
[58.] *Canadian Institute for Health Research, Ottawa, ON;*
[59.] *Department of Medical Biophysics, University of Toronto, Toronto, ON;*
[60.] *Department of Ophthalmology and Visual Sciences, Research Institute of the McGill University Health Center, Montreal, QC;*
[61.] *Department of Medical Imaging, University of Toronto, Toronto, ON;*
[62.] *Neuropsychology and Cognitive Health Program, Baycrest Health Sciences; Department of Psychology, University of Toronto, Toronto, ON*
