# Supplementary material for: Contribution of rare variant associations to neurodegenerative disease presentation
Source: NPJ Genom Med. 2021 Sep 28;6:80. doi: 10.1038/s41525-021-00243-3 (PMC8478934; doi:10.1038/s41525-021-00243-3)
Supplement: Supplementary file 2 — Reporting Summary [file 41525_2021_243_MOESM2_ESM.pdf]

nature research

Corresponding author(s): Dr. Robert Hegele and Allison Dilliott

Last updated by author(s): Aug 24, 2021

Reporting Summary

Nature Research wishes to improve the reproducibility of the work that we publish. This form provides structure for consistency and transparency in reporting. For further information on Nature Research policies, see our Editorial Policies and the Editorial Policy Checklist.

Statistics

For all statistical analyses, confirm that the following items are present in the figure legend, table legend, main text, or Methods section.

n/a	Confirmed	
☐	☒	The exact sample size (n) for each experimental group/condition, given as a discrete number and unit of measurement
☒	☐	A statement on whether measurements were taken from distinct samples or whether the same sample was measured repeatedly
☐	☒	The statistical test(s) used AND whether they are one- or two-sided<br>*Only common tests should be described solely by name; describe more complex techniques in the Methods section.*
☐	☒	A description of all covariates tested
☐	☒	A description of any assumptions or corrections, such as tests of normality and adjustment for multiple comparisons
☐	☒	A full description of the statistical parameters including central tendency (e.g. means) or other basic estimates (e.g. regression coefficient) AND variation (e.g. standard deviation) or associated estimates of uncertainty (e.g. confidence intervals)
☐	☒	For null hypothesis testing, the test statistic (e.g. F, t, r) with confidence intervals, effect sizes, degrees of freedom and P value noted<br>*Give P values as exact values whenever suitable.*
☒	☐	For Bayesian analysis, information on the choice of priors and Markov chain Monte Carlo settings
☒	☐	For hierarchical and complex designs, identification of the appropriate level for tests and full reporting of outcomes
☒	☐	Estimates of effect sizes (e.g. Cohen's d, Pearson's r), indicating how they were calculated

Our web collection on statistics for biologists contains articles on many of the points above.

Software and code

Policy information about availability of computer code

Data collection	Next-generation sequencing data was processed using a bioinformatics workflow in CLC Bio Genomics Workbench v10 (CLC Bio, Aarhus, Denmark) and annotated using VarSeq® (Golden Helix, Bozeman, MT, United States).
Data analysis	The Optimal Sequence Kernal Association Test was performed using the Exhautomate package, available at: https://github.com/exautomate/Exautomate-Core. Remaining statistical analyses were performed using R statistical software 3.6.0 in R Studio 1.1.463 and data visualization was performed using the ggplot2 R package (v3.3.s).

For manuscripts utilizing custom algorithms or software that are central to the research but not yet described in published literature, software must be made available to editors and reviewers. We strongly encourage code deposition in a community repository (e.g. GitHub). See the Nature Research guidelines for submitting code & software for further information.

Data

Policy information about availability of data

All manuscripts must include a data availability statement. This statement should provide the following information, where applicable:

- Accession codes, unique identifiers, or web links for publicly available datasets
- A list of figures that have associated raw data
- A description of any restrictions on data availability

In accordance with the Ontario Neurodegenerative Disease Research Initiative (ONDRI) with the Ontario Brain Institute, all baseline data from ONDRI are available upon request at https://www.braincode.ca/. All data have been de-identified. To gain access to the data, an account request must be made to help@braincode.ca. This process requires the applicant to provide their contact information and association, which are then verified. Data access will require the completion of a Data Access Request, which will be provided following the initial account request. Further details regarding data access can be found at https://www.braincode.ca/content/getting-started. The data are not available publicly outside of the Brain-CODE portal due to information that could compromise the privacy of the research

participants.

Field-specific reporting

Please select the one below that is the best fit for your research. If you are not sure, read the appropriate sections before making your selection.

☒ Life sciences ☐ Behavioural & social sciences ☐ Ecological, evolutionary & environmental sciences

For a reference copy of the document with all sections, see nature.com/documents/nr-reporting-summary-flat.pdf

Life sciences study design

All studies must disclose on these points even when the disclosure is negative.

Sample size	In total, we enrolled 520 neurodegenerative disease participants into the ONDRI study. One individual did not provide a blood sample for DNA isolation, leaving the total case sample size as 519. Additionally, 189 control DNA samples were obtained from the GenADA study. Following genetics-based ancestral outlier analysis, 396 ONDRI cases and 164 controls remained for use in the rare variant association analyses.
Data exclusions	One ONDRI participant was excluded from the analysis, as no blood sample was obtained upon enrollment. Based on the genetics-based outlier analysis, 123 ONDRI cases and 25 controls were excluded from the rare variant association analyses.
Replication	Results were not replicated due to a lack of replication cohort comprised of the six diagnoses under study. This limitation was addressed in the discussion, with suggestion for future replication.
Randomization	Randomization was not relevant for our study, as participants were not given any experimental treatments.
Blinding	Blinding was not relevant to our study, as participants were enrolled as part of an observational cohort study.

Reporting for specific materials, systems and methods

We require information from authors about some types of materials, experimental systems and methods used in many studies. Here, indicate whether each material, system or method listed is relevant to your study. If you are not sure if a list item applies to your research, read the appropriate section before selecting a response.

Materials & experimental systems

n/a	Involved in the study	
☒	☐	Antibodies
☒	☐	Eukaryotic cell lines
☒	☐	Palaeontology and archaeology
☒	☐	Animals and other organisms
☐	☒	Human research participants
☒	☐	Clinical data
☒	☐	Dual use research of concern

Methods

n/a	Involved in the study	
☒	☐	ChIP-seq
☒	☐	Flow cytometry
☒	☐	MRI-based neuroimaging

Human research participants

Policy information about studies involving human research participants

Population characteristics	The ONDRI neurodegenerative disease cases had a mean age of 68.6 years and a male-to-female ratio of 341:172. The elderly, cognitively normal control cohort had a mean age of 74.0 years and a male-to-female ratio of 77:112. Statistical analyses were corrected for age and sex. We sequenced all cases and controls using a customized next-generation sequencing panel, ONDRISeq, that covers the exonic regions of 80 genes previously associated with neurodegenerative disease. Leveraging the common genetic data obtained using ONDRISeq, we performed outlier analysis to ancestry match the case and control cohorts. All participants in the ONDRI cohort had one of the following diagnoses: Alzheimer's disease, amyotrophic lateral sclerosis, cerebrovascular disease, frontotemporal dementia, mild cognitive impairment, or Parkinson's disease.
Recruitment	Participants were recruited at tertiary care clinics located at 14 health science centres across Ontario based on disease specific inclusion and exclusion criteria, which are cited within the manuscript. Due to the demands of the assessments included in the ONDRI study, enrolled participants tended to have more mild forms of disease, potentially causing a bias towards sporadic neurodegenerative disease cases. However, sporadic neurodegenerative disease cases are generally more common, and so our cohort may accurately reflect the general population. We do not anticipate that recruitment introduced bias to our results.
Ethics oversight	Study ethics approval was obtained from the Research Ethics Boards at Baycrest Centre for Geriatric Care (Toronto, Ontario, Canada); Centre for Addiction and Mental Health (Toronto, Ontario, Canada); Elizabeth Bruyère Hospital (Ottawa, Ontario, Canada); Hamilton General Hospital (Hamilton, Ontario, Canada); McMaster (Hamilton, Ontario, Canada); London Health Sciences Centre (London, Ontario, Canada); Parkwood Hospital (London, Ontario, Canada); St Michael's Hospital (Toronto, Ontario, Canada); Sunnybrook Health Sciences Centre (Toronto, Ontario, Canada); The Ottawa Hospital (Ottawa, Ontario, Canada); and University Health Network-Toronto Western Hospital (Toronto, Ontario, Canada).

Note that full information on the approval of the study protocol must also be provided in the manuscript.
